# Mitochondrial oxidative phosphorylation in cutaneous melanoma

**DOI:** 10.1038/s41416-020-01159-y

**Published:** 2020-11-18

**Authors:** Prakrit R. Kumar, Jamie A. Moore, Kristian M. Bowles, Stuart A. Rushworth, Marc D. Moncrieff

**Affiliations:** 1 Bob Champion Research and Education Building, Norwich Medical School, University of East Anglia, Norwich, UK; 2 Department of Haematology, Norfolk and Norwich University Hospital, Norwich, UK; 3 Department of Plastic and Reconstructive Surgery, Norfolk and Norwich University Hospital, Norwich, NR4 7UY UK

**Keywords:** Melanoma, Cancer metabolism

## Abstract

The Warburg effect in tumour cells is associated with the upregulation of glycolysis to generate ATP, even under normoxic conditions and the presence of fully functioning mitochondria. However, scientific advances made over the past 15 years have reformed this perspective, demonstrating the importance of oxidative phosphorylation (OXPHOS) as well as glycolysis in malignant cells. The metabolic phenotypes in melanoma display heterogeneic dynamism (metabolic plasticity) between glycolysis and OXPHOS, conferring a survival advantage to adapt to harsh conditions and pathways of chemoresistance. Furthermore, the simultaneous upregulation of both OXPHOS and glycolysis (metabolic symbiosis) has been shown to be vital for melanoma progression. The tumour microenvironment (TME) has an essential supporting role in promoting progression, invasion and metastasis of melanoma. Mesenchymal stromal cells (MSCs) in the TME show a symbiotic relationship with melanoma, protecting tumour cells from apoptosis and conferring chemoresistance. With the significant role of OXPHOS in metabolic plasticity and symbiosis, our review outlines how mitochondrial transfer from MSCs to melanoma tumour cells plays a key role in melanoma progression and is the mechanism by which melanoma cells regain OXPHOS capacity even in the presence of mitochondrial mutations. The studies outlined in this review indicate that targeting mitochondrial trafficking is a potential novel therapeutic approach for this highly refractory disease.

## Background

Melanoma is the most aggressive, deadly form of skin cancer^1^—despite accounting for only 5% of cases, it constitutes the main cause of deaths from skin cancer.^2^ It is also one of the fastest growing cancers worldwide,^2^ with the UK reporting 16,000 new cases every year.^3^ Along with the long-standing global trend of incidence rise,^4^ worldwide mortality rates are expected to increase from 61,850 in 2016 to 108,630 by 2040.^5^

Melanoma is highly curable when limited to the primary site;^6^ metastatic melanoma, however, confers a poor prognosis of a median survival of 6 months.^7^ Current systemic therapies in patients with metastatic melanoma have a varied response rate, and tumour resistance develops rapidly in the majority of patients.^6,8–10^ Further research is therefore required to understand the pathophysiology of this highly refractory disease, in the context of the role of metabolism (oxidative phosphorylation and/or glycolysis) in melanoma, and the interaction of melanoma with the tumour microenvironment (TME), which supports its survival and proliferation, and contributes to drug resistance.

Primary cutaneous melanoma comprises a distinctly heterogeneous population of both cancerous and noncancerous cells,^11,12^ including fibroblasts, adipocytes and other niche cells such as mesenchymal stromal cells (MSCs), which make up the extracellular matrix, endothelial cells of the microvasculature and immune cells.^11–13^ In addition to the cellular component of the tumour microenvironment (TME), the noncellular component consists of several growth factors, chemokines and cytokines.^14^ Melanoma cells can manipulate the close association between themselves and the TME to facilitate tumour progression, invasion and metastasis.^15,16^ Currently, immune cells in the TME have been the focus of much interest in an attempt to understand how an immunosuppressive microenvironment that allows for proliferation, growth and invasion of melanoma is created,^10^ while, by contrast, relative little research has been carried out on the role of MSCs in the TME in melanoma growth.

In this review, we explore the symbiotic relationship between melanoma and MSCs and the ensuing metabolic advantage conferred on melanoma. We begin by describing the metabolism of melanoma and metabolic plasticity in melanoma cells before introducing metabolic symbiosis with MSCs and outlining potential mechanisms of transfer of mitochondrial DNA from MSCs to melanoma to facilitate oxidative phosphorylation.

## MSCs in the time

MSCs—spindle-shaped cells that are present in bone marrow, adipose, skin, umbilical cord, blood and various other tissues^17–20^—are highly proliferative and can differentiate into various cells such as osteoblasts, chondrocytes and adipocytes.^17–19^ These properties, in addition to their ability to home towards injured tissue, can be exploited by melanoma, which, like many other solid cancers, behaves like tissues that do not heal:^21,22^ increasing evidence has shown that, like a chronic, nonhealing wound, melanoma secretes chemoattractants,^23^ similar to those used in inflammatory signalling pathways,^24^ to attract and direct MSCs towards the tumour sites and form part of the TME to contribute towards tumour progression, invasion and metastasis.^23,25–28^

### MSCs and melanoma growth

A positive effect for MSCs on tumour incidence was first demonstrated by co-injecting allogeneic mice with B16 melanoma cells and MSCs: not only was the incidence of tumour formation 100% when the melanoma cells and MSCs were injected together versus 0% in the control group,^29,30^ but also the onset of tumour formation was faster when soluble MSC-derived factors were added.^31^ Kucerova et al. demonstrated this increased tumour incidence and growth using the human melanoma cell line A375 and human MSCs, as well as showing that this increase was dependent on the dose of MSCs.^32^ MSCs also abrogated tumour latency in vivo for low numbers of cells that would otherwise not normally produce tumours if injected alone.^32^ Furthermore, MSCs were shown to protect melanoma cells from sustaining cellular stress in response to systemic treatment, such as doxorubicin, and cytotoxicity by inhibiting apoptosis. Notably, the effect of MSCs on tumour initiation was reported in experiments using low volumes of A375 melanoma cells, mimicking minimal residual disease that is common following radiotherapy treatment. Together, these data demonstrate the pro-oncogenic role of MSCs on melanoma growth.

### Additional pro-oncogenic roles of MSCs

MSCs also display various other pro-oncogenic behaviours, which are outlined here but not covered in detail as they are not the focus of this review. MSCs have been reported to increase the motility and invasiveness of melanoma by communicating with melanoma-derived exosomes, to manipulate melanoma cells towards a more metastatic phenotype via the process of epithelial-to-mesenchymal transition (EMT)^33^ and by increasing the porosity of blood vessels, thereby facilitating tumour migration.^33^ Current reports have demonstrated the ability of MSCs to support neovascularisation in a mouse model of melanoma through the secretion of proangiogenic factors.^27,28,34^ Kucerova et al. demonstrated enhanced melanoma growth as a result of this proangiogenic cellular milieu created by the mutual crosstalk between melanoma and MSCs.^32^ In addition to the secretion of various factors, Vartanian et al. provided direct evidence that melanoma can educate MSCs to engage in vasculogenic mimicry, a process in which MSCs adopt certain endothelial-cell-like properties to directly contribute to the formation of the tumour vasculature.^28^ Several studies have also demonstrated the ability of MSCs to differentiate into carcinoma-associated fibroblasts (CAFs),^28,34^ a key cellular component of the growth-supporting TME, aiding the formation of the stem-cell niche and promoting stemness in the tumour.^23,35^ Not only do these CAFs and MSCs promote tumour growth, but they have also been shown to have immunomodulatory functions—for example, reducing T-cell proliferation and the number of tumour-infiltrating T and B cells in vivo, and producing cytokines—thereby creating a highly effective immunosuppressive TME for melanoma proliferation.^23,36^

### MSC—melanoma symbiosis confers metabolic advantage

The processes of MSC-mediated tissue repair, which involves activation, migration and homing to TME, and MSC differentiation and subsequent secretion of factors (by both melanoma cells and MSCs) produce a strong pro-oncogenic symbiotic relationship between MSCs and melanoma cells.^21^ This symbiotic relationship provides a metabolic advantage that is effective for melanoma proliferation and metastasis.^37–39^ Given the significance of metabolism in melanoma, supported by the growing evidence of its impact on the efficacy of current systemic therapies for this highly refractory disease,^38^ below we explore the symbiotic relationship between MSCs and melanoma, and how it might arise.

## Metabolism of melanoma

Due to its significant influence on all aspects of tumorigenesis, metabolic reprogramming has been widely accepted as one of the hallmarks of cancer.^40^ Determining the biochemical pathway that melanoma cells use for energy production allows researchers to understand the influence of metabolism on the symbiotic relationship between melanoma and MSCs and its corresponding pro-oncogenic role.^41^

### Glycolysis in melanoma

In the 1920s, Warburg reported that, even in presence of oxygen, cancer cells take up glucose for glycolysis.^42^ This preferential method for energy production adopted by cancers was termed ‘aerobic glycolysis’ (also known as the Warburg effect) and was demonstrated to provide ATP necessary for survival and proliferation of the tumour.^43^

Melanoma has been demonstrated to be associated with a glycolytic phenotype.^44,45^ Aerobic glycolysis in melanoma cells is driven by a multitude of factors, including activation of oncogenes, the presence of a hypoxic TME and an absence of tumour suppressors.^46^ Approximately 50–60% of melanomas contain a *BRAF* gene mutation,^47^ the most frequent of which (BRAFV600E, accounting for 90% of BRAF mutations^48^ and rendering the gene product B-Raf constitutively active^49^) has been shown to be associated with higher glucose uptake and subsequent glycolysis.^50^ B-Raf activates the mitogen-activated protein kinase (MAPK) pathway, which promotes hypoxia-inducible factor 1α [HIF1α (master regulator of glycolysis)], resulting in an increase in glycolysis.^50^ Furthermore, B-Raf inhibits microphthalmia-associated transcription factor (MITF) and peroxisome proliferator-activated receptor-γ coactivator 1α (PGC-1α), thereby inhibiting oxidative phosphorylation (OXPHOS).^43,51,52^ OXPHOS is the main pathway for energy production in mitochondria via aerobic respiration. Providing direct evidence for this B-Raf mediated upregulation of aerobic glycolysis, Hall et al.^44^ demonstrated a 14–16-fold higher extracellular acidification rate (ECAR, resulting from respiratory and glycolytic acidification) in melanoma cells compared with melanocytes. Furthermore, treatment with the glycolysis inhibitor 2-deoxy-D-glucose (2-DG) induced a significant drop in ATP production by melanoma cells, causing them instead to revert to OXPHOS for energy production. Analysis of the ECAR (a surrogate marker for glycolysis) and oxygen consumption rate (OCR; a surrogate marker for OXPHOS) in these cells uncovered a lower OCR/ECAR ratio, indicating the upregulation of glycolysis rather than low oxygen consumption or lower OXPHOS capacity. In fact, the absolute OCR values were higher in melanoma cell lines compared with melanocytes, with corresponding high OXPHOS enzyme activity. Therefore, although glycolysis is upregulated in melanoma, OXPHOS also plays a role.

### Oxidative phosphorylation in melanoma

Whether a specific metastatic lesion relies on either glycolysis or OXPHOS depends upon the heterogeneity of individual tumour types.^53^ Tumours behave individually, with each cancer demonstrating its own metabolic properties.^46,53^ To add further complexity, even within an individual tumour, the constituent cells can be heterogeneous, displaying different energy metabolic phenotypes.^46^ For example, large B cell lymphomas can be split into a low OXPHOS subset and a high OXPHOS subset; the latter subset show an upregulation of mitochondrial electron transport chain components.^54^ While many melanomas have an aerobic glycolytic phenotype, a subset has been shown to present with higher OXPHOS phenotype.^38,52^ Fischer et al. have identified that 35–50% of BRAF-mutant and wild-type cell lines and patient samples can be categorised into this subset,^38^ indicating that a significant proportion of melanoma cells present with a higher OXPHOS phenotype. PGC-1α is a member of a family of transcriptional coactivators that play a central role in the regulation of cellular energy metabolism and mitochondrial biogenesis.^55^ Regulatory mechanisms to suppress OXPHOS mediated via the PGC-1α pathway fail to occur in high OXPHOS melanomas.^38^ Higher PGC-1α levels are correlated with poorer survival in melanoma patients.^52^ The PGC-1α-driven high OXPHOS subset demonstrated an improved tolerance to the damaging effects of reactive oxygen species (ROS), indicating their increased ability to survive under conditions of oxidative stress.^52^ In vivo experiments in mice demonstrated that PGC-1α knockdown resulted in reduced metastasis of melanoma,^56^ highlighting the pro-oncogenic role of OXPHOS in melanoma progression and metastasis.

### OXPHOS and glycolysis in melanoma

Ho et al*.*^57^ suggested that both OXPHOS and glycolysis play a significant role in the progression of melanoma and generation of ATP. They discovered two patient populations within their melanoma cohorts: one with high serum levels of lactate dehydrogenase (LDH) and one with normal serum LDH levels. The high serum LDH population had a corresponding high ECAR, suggesting that glycolysis was the predominant metabolic pathway. By contrast, in the normal serum LDH population, the tumours demonstrated elevation of several OXPHOS enzymes and higher OCR, indicating that OXPHOS was the predominant metabolic pathway. However, it is important to note that, although high serum LDH levels are associated with poor prognosis in metastatic melanoma patients,^58^ serum LDH levels might not necessarily always be a marker of tumour-associated increased cell turnover, as patients can have high LDH levels and perform poor clinically due to other factors, such as tissue damage, severe infections and renal failure.^59^ The OCR rates were higher in both populations of melanoma patients, as well as in melanoma samples from patient tumour biopsy samples and cell lines in culture, compared with normal melanocytes. Thus, OXPHOS and glycolysis both play a significant role in melanoma metabolism.^60,61^

## Metabolic plasticity

Although it is simpler to categorise melanoma into a glycolytic or OXPHOS phenotype, an increasing body of evidence suggests that the nature of metabolic phenotypes in melanoma is dynamic—this is termed ‘metabolic plasticity’.^38,40^ Jose et al*.*^40^ demonstrated that the metabolic phenotype of melanoma is not fixed during tumorigenesis and, in fact, melanoma has a ‘hybrid’ glycolysis/OXPHOS metabolic phenotype, intuitively conferring selective advantages on tumour cells.^45^ Importantly, this hybrid phenotype provides tumour cells with the flexibility to use different energy sources to meet their bioenergetic needs in the different and changing TME.^62^ In a glucose-deprived environment, tumour cells are metabolically reprogrammed towards elevated levels of OXPHOS with decreased glycolysis, whereas in hypoxic conditions, the melanoma cells preferentially use glycolysis, uncoupling from the TCA cycle and attenuating mitochondrial respiration.^45^

Metabolic plasticity confers on melanoma cells not only the ability to adapt to harsh TME conditions but also a degree of chemoresistance, thereby providing a survival advantage in treatment-induced conditions.^38^ The use of targeted systemic therapy such as BRAF inhibitors (e.g. vemurafenib) to target BRAFV600E is associated with a switch from glycolysis to OXPHOS.^63^ Similarly, Haq et al. demonstrated that treatment with MAPK inhibitors resulted in increased PGC-1α-driven OXPHOS.^51^ Notably, an increase in PGC-1α-driven OXPHOS is observed in 30–50% of *BRAF*-mutant melanomas with *de novo* and acquired resistance to MAPK inhibitors^64^and, in these circumstances, PGC-1α knockdown resulted in reduced tumour growth.^64^ This metabolic switch from glycolysis to OXPHOS and the corresponding adaptive resistance was observed in patients treated with inhibitors of BRAF or MEK (MAPK and ERK kinase; an upstream activator of MAPK), alongside increased mitochondrial content, mitochondrial activity and mitochondrial oxidative capacity.^37,65–68^ These observations highlight the ability of melanoma to redirect the metabolic phenotypes to confer multiple pathways of chemoresistance. Collectively, it is clear that metabolic plasticity confers a significant survival advantage on cancer cells.

## Metabolic symbiosis

Within melanoma tumours, the extent of tissue perfusion and oxygenation depends on the location and physical distance of the tumour cells from the local vasculature.^57^ Accordingly, melanoma cells located in the poorly perfused centre of tumour masses are more likely to be predominantly dependent on glycolysis, whereas tumour cells closer to the vasculature at the periphery are more likely to be dependent on OXPHOS.^57^ It has, however, been proposed that these two spatially distinct cell populations might be linked, such that the end products from glycolysis (e.g. lactate) are utilised to feed into the TCA cycle for OXPHOS.^57^

**Fig. 1:**
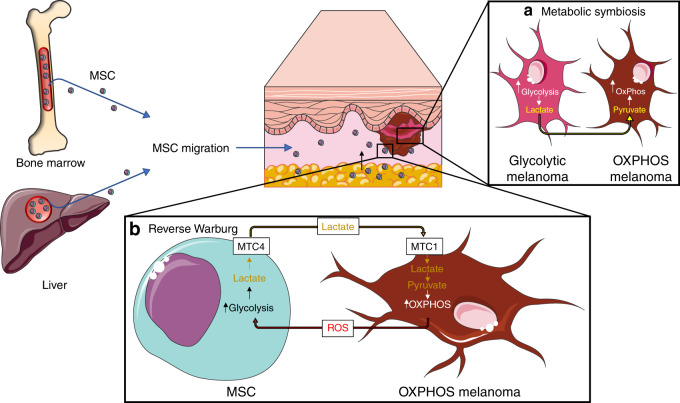
Metabolism in melanoma. **a** The smooth co-operation of OXPHOS and glycolysis in the two spatially distinct melanoma cell populations (melanoma cells in the centre that use glycolysis versus melanoma cells in periphery that use OXPHOS mainly for energy production) promotes melanoma initiation, growth and metastasis of melanoma through metabolic symbiosis, whereby the waste products from glycolysis are used to feed into the TCA cycle for OXPHOS in melanoma cells in the periphery. **b** Mesenchymal stromal cells (MSCs) migrate from the bone marrow and liver towards the melanoma, where they are then manipulated by tumour cells to produce lactate and other macromolecules via glycolysis, for use by melanoma cells that mainly use OXPHOS in the peripheral part of the tumour (Reverse Warburg).

Indeed, Ho et al*.*^57^ reported that, although melanoma patients showed high levels of serum LDH, monocarboxylate transporters MCT4, (the principal transporter for lactate efflux^69^ and a downstream effector of hypoxia-inducible factor (HIF)-1a,^70^) indicating that the melanoma cells predominantly used glycolysis for energy production, the serum lactate levels remained constant. Although it is plausible that the lactate levels might not be affected by the tumour, the above data demonstrating upregulation of glycolysis make it more likely that the lactate levels remain unchanged due to other processes. Ho et al. suggested that increased levels of lactate resulting from glycolysis are taken up by the metabolically symbiotic melanoma cells that use OXPHOS as their primary mechanism of energy production. When enzymes associated with OXPHOS and glycolysis were both expressed at higher levels, it was evident that OXPHOS and glycolysis were both upregulated in melanoma, compared with normal tissues, demonstrating a further stage to metabolic plasticity.^57^ This co-operation of both OXPHOS and glycolysis occurring at the same time has been coined ‘metabolic symbiosis’ (Fig. 1a). Several papers^71^ have reported this phenomenon and demonstrated its importance in melanoma initiation, growth and metastasis.

## The reverse warburg effect

In vivo work carried out over the past decade has demonstrated that metabolic reprogramming involves not only cancer cells but also the MSCs and CAFs in the TME.^62,72^ Whereas the Warburg effect refers to glycolysis being the preferential method of energy production in tumour cells, according to the ‘Reverse Warburg’ effect, tumour cells, by secreting ROS (by-product of OXPHOS melanoma cells), stimulate cells in the surrounding TME to undergo aerobic glycolysis, resulting in the secretion of metabolites, such as lactate, into the TME via MCT4.^73^ These metabolic intermediates can be taken up by tumour cells, via MCT1, to feed into the TCA cycle for OXPHOS-mediated energy production,^74^ similar to the situation in metabolic symbiosis outlined above. Loss of Cav-1, a protein involved in endocytosis and vesicular transport, in TME cells results in a positive-feedback loop of oxidative stress in these cells, consequently increasing OXPHOS in tumour cells.^62^ This Reverse Warburg effect was initially reported in a variety of cancers^38^ but is as yet to be fully elucidated in melanoma. However, taking together the use of OXPHOS in the periphery of melanomas, the metabolic symbiosis reported earlier, and Ho et al.’s findings of increased expression of MTC1 and MTC4 in melanoma,^57^ it is likely that the Reverse Warburg effect occurs in the TME of melanoma (Fig. 1b).

The above research has demonstrated that the oncological hallmark of altered metabolism is not only due to the regulation for growth, but can also be primary cause for tumour initiation, progression, metastasis and chemoresistance. Due to the heterogeneic dynamism (metabolic plasticity) between glycolysis and OXPHOS of melanoma, the effective blockade of OXPHOS (e.g. using inhibitors of mTORC1) as well as glycolysis (e.g. MAPK pathway inhibitors) has been shown to resensitise melanomas that are resistant to inhibitors of BRAF and other MAPK pathway components, and thus to be a promising form of treatment.^64,75^ Previous work has shown that upregulation of aerobic glycolysis in tumour cells is due to the presence of mitochondrial DNA (mtDNA) mutations, which were assumed to impair OXPHOS capacity. However, several papers have demonstrated that these mtDNA mutations do not necessarily equate to a compromise in OXPHOS capacity. Conversely, although cancer cells retain OXPHOS capacity, they can also possess mtDNA mutations due to damaging effects of higher ROS secretion in mitochondria from inefficient repair mechanisms, close proximity and vulnerability of mtDNA.^43,46,50,76^ Consequently, further research was carried out to discover why melanoma cells with mtDNA mutations still possessed the capacity to use OXPHOS for energy production, as well as to develop more effective OXPHOS therapies against melanoma.

## Mitochondrial transfer

In 2010, Berridge and Tan^77^ designed a model of B16 melanoma cell lines with severe mtDNA damage caused by the mitochondrial gene deletion ρ0 to investigate mitochondrial OXPHOS. The authors observed that not only did the ρ0 cells grow at half the rate of their parental cells in vitro, but they also underwent delayed primary subcutaneous melanoma growth and reduced lung metastasis formation in mouse models in vivo, compared with B16 parental cells.^77^ At the time these studies were carried out, this delay was suggested to be due to the time taken to adapt to auxotrophic requirements and local microenvironmental conditions. However, a series of in vitro experiments demonstrating mitochondrial trafficking in other cancers (Table 1) as a prerequisite for aerobic respiration, tumour growth, metastasis and chemoresistance^78–88^ implied that the delay might be the result of mitochondrial trafficking from MSCs in the TME to tumours. Additional investigations in other cancers into the mechanisms and stimuli behind mitochondrial trafficking, such as NAPDH-oxidase-2-driven and CD38-driven in acute myeloid leukaemia and multiple myeloma, respectively, have led to the development of effective therapeutic agents targeting mitochondrial trafficking, with demonstrated effective tumour regression.^83,84,89^

**Table 1:** Mitochondrial transfer from the TME to cancer cells.

Recipient tumour (cell type)	Mouse (M)/human (H) of tumour	Donor	In vitro/in vivo	Reference
**Other cancers**				
*Lung adenocarcinoma (A459)*	H	Human MSCs	In vitro	^87^
*Osteosarcoma (143B)*	H	Human MSCs	In vitro	^81^
*Ovarian carcinoma (SKOV3, OVCAR3)*	H	Human MSCs, Endothelial cells	In vitro	^86^
*Breast carcinoma (MDA-MB231, MCF7)*	H	Human MSCs, Endothelial cells	In vitro	^86^
*Lung adenoma (mLA-4)*	M	Human MSCs	In vitro	^78^
*Osteosarcoma (143B)*	H	Human MSCs	In vitro	^82^
*Breast carcinoma (mda-mb-231)*	H	Human MSCs	In vitro	^80^
*Breast carcinoma (4T1)*	H	Mouse MSCs	In vivo	^88^
*Acute myeloid leukaemia (HL-60, Kasumi-1, KG-1, MOLM-14, NB-4, SKM-1, THP-1, and U-937)*	H	Human MSCs	In vivo	^85^
*Acute myeloid leukaemia (Primary)*	H	Human MSCs	In vivo	^84^
*Multiple myeloma (Primary, MM1s, U266)*	H	Mouse MSCs	In vivo	^83^
*Acute lymphoblastic leukaemia (REH, SD1, SEM, and TOM1)*	H	Human MSCs	In vivo	^79^
**Melanoma**				
*(B16)*	M	Mouse MSCs	In vivo	^88^
*(B16)*	M	Mouse MSCs	In vivo	^90^

### Mitochondrial transfer in melanoma

Consistent with the results obtained in other tumours, Tan et al*.*^88^ subsequently demonstrated, in 2015, that the delay in melanoma tumour growth when B16ρ0 cells were injected in NOD/SCID mice was due to the time taken for these cells to acquire mtDNA from the TME in vivo. In 2017, Dong et al*.*^90^ demonstrated that the tumours that grew from injected B16ρ0 cells in vivo, after a delay, contained host TME mtDNA (confirmed via single-cell droplet PCR methods), and that the B16ρ0 cells had acquired mitochondria from host MSCs by the presence of double-positive cells when B16ρ0 cells with nuclear-targeted blue fluorescent protein were injected into C57BL/GN mice with red fluorescent mitochondria in mouse MSCs.

The primary role of mitochondria is to produce energy via OXPHOS,^91^ and mtDNA encodes peptides that are essential for this task.^92^ Accordingly, Dong et al. demonstrated that the injected B16ρ0 cells that gained mtDNA subsequently contained mtDNA-encoded proteins and fully assembled respirasomes, with a higher OCR and increased ATP production than injected B16ρ0 cells that failed to gain mtDNA. These results demonstrated that the mtDNA transferred to the B16ρ0 cells was functional and conferred similar OXPHOS respiration rates and respiration recovery to those of their parental B16 cells.

Finally, Dong et al*.*^90^ provided direct evidence for the requirement of OXPHOS respiration mediated by mtDNA transfer from MSCs to melanoma cells in tumour formation. B16ρ0 cells with OXPHOS respiration suppressed (via inhibition of the catalytic subunits of CI and CII) formed tumours with an even longer lag period of 15–40 days compared with B16ρ0 cells without OXPHOS suppressed, in vivo. A similar pattern was observed for parental B16 cells with OXPHOS knocked down.^90^ This indicates a shift in viewpoint regarding cancer metabolism, with mitochondrial DNA mutations not compromising OXPHOS capacity. These results collectively demonstrate that melanoma cells gain mtDNA from the MSCs and their subsequent rapid OXPHOS recovery is a prerequisite for driving efficient tumour formation. Although mitochondrial trafficking in melanoma has only been shown in the murine B16 cell line, the extensive experiments mentioned above, coupled with the importance of mitochondrial trafficking demonstrated in several other human cancers, make it very plausible that mitochondrial trafficking plays a vital role in human melanoma progression. A major gap in the literature therefore exists, and further experiments are required to demonstrate role of mitochondrial trafficking in other cell lines and human melanoma.

Despite intracellular transfer of mtDNA being the most likely transfer mechanism, other possible explanations for mtDNA acquisition and respiration recovery have been suggested. First, it is plausible that a few tumour cells with mitochondria and mtDNA replicate their mtDNA and proliferate at much faster rate than tumour cells without mtDNA, and that the tumour cells without mtDNA might then be progressively removed by autophagy. However, markers for autophagy, such asLC3A11 protein, were lower in B16ρ0 cells compared with B16 cell lines, suggesting that this is not the case.^90^ Another possible explanation is the presence of B16ρ0 cells that contained residual undetectable mtDNA. However, this theory was rejected by Dong et al., who used assays that were able to detect heteroplasmy down to 0.5%. The absence of mtDNA in ρ0 cells was further reinforced by confocal microscopy analysis, and the absence of any latent respirasomes/super-complexes prior to mtDNA acquisition was shown via native blue gel electrophoresis. Thus, the only plausible mechanism of mtDNA acquisition in tumour cells is thought to be transfer from host TME.

## Mechanism of mtDNA transfer

**Fig. 2:**
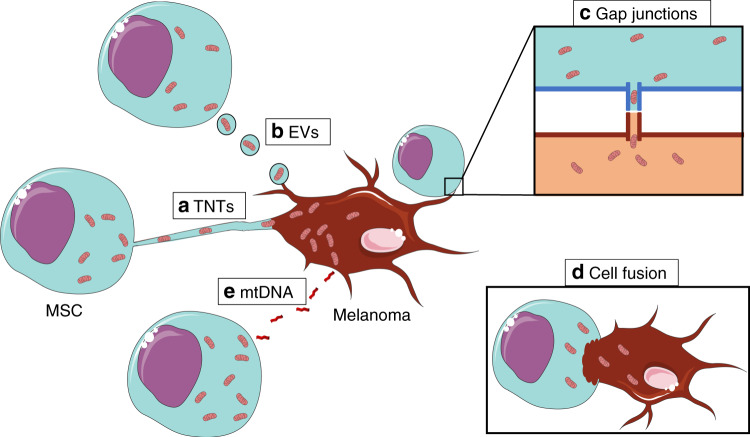
Mechanisms of mtDNA transfer. **a** Tunnelling nanotubules (TNTs), **b** microvesicles and **c** gap junctions, as well as other plausible mechanisms that require further research, such as **d** cell fusion and **e** direct mtDNA secretion into extracellular media.

Studies carried out over the past 15 years have demonstrated that mitochondria can cross cell boundaries and be transferred horizontally between cells.^93^ The main mechanisms of mtDNA transfer from MSCs to tumour cells are tunnelling nanotubules (TNTs), microvesicles and gap junctions, although other plausible mechanisms exist that require further research, such as cell fusion and direct mtDNA secretion into extracellular media^93–95^ (Fig. 2).

### Tunnelling nanotubules

Tunnelling nanotubes (TNTs) are filopodial extensions (bundles of rod-like shaped parallel actin filaments) of cell cytoplasm that connect two cells via open-ended channels^96,97^ (Fig. 2a). TNTs have been shown to facilitate the transfer of biomaterial such as cellular organelles, cytoplasmic molecules and membrane molecules between cells.^97^ Koyangi et al. were the first to document (in 2005) whole mitochondrial transfer through TNTs from cardiomyocytes to endothelial progenitor cells;^98^ mitochondrial transfer into melanoma via TNTs was not demonstrated until 2017.^90^

### Extracellular vesicles (EVs)

mtDNA can also be horizontally transferred through the movement of mitochondrial-derived products or intact mitochondria in EVs—exosomes or microvesicles, respectively^93^ (Fig. 2b). Exosomes are small (30–100 nM in diameter) membrane-encompassed vesicles formed in the endosomal pathway.^94,99,100^ During the endosomal pathway, segments of endosomal membrane bud inside the endosome as a collection of intraluminal vesicles (ILVs) to form multivesicular bodies (MVBs).^94^ These MVBs move to the cell’s surface plasma membrane release ILVs (exosomes) externally into the extracellular media.^94,101^ In contrast, microvesicles, largest EVs (50–1000 nM in diameter),^99^ are formed directly from external budding and fission of the plasma membrane of the cell into the extracellular media.^94,102,103^ Guescini et al. demonstrated the potential of exosomes as vectors for mitochondrial transfer in glioblastoma and astrocyte cells, which routinely secrete EVs into the intercellular space.^104^ Isolation of these purified EVs demonstrated the presence of mtDNA and absence of nuclear DNA. Furthermore, high mtDNA levels and mitochondrial proteins were shown to be present in exosomes released into the intercellular media by skeletal muscle cells.^105^ Although these papers implied that EVs could function as mtDNA carriers, Islam et al*.*^106^ were the first to demonstrate mitochondrial transfer from MSCs to lung alveolar epithelial cells via EVs in vivo. Furthermore, Sinclair et al.^95^ demonstrated that mitochondrial trafficking was reduced by 34.7% after inhibition of endocytosis of EVs into lung epithelial cells. These results provide in vivo evidence for the transfer of mitochondria from MSCs to tumour cells via EVs, although transfer from MSCs to melanoma by this means has not yet been reported.

### Gap junctions

Gap junctions are intercellular channels composed of two connexons, joined together in the intercellular space, that directly connect the cytoplasm of two different cells^107^ (Fig. 2c). Whereas TNTs facilitate long-distance communication, gap junctions promote close cell-to-cell communication.^107^ Islam et al*.*^106^ demonstrated gap-junction-mediated mitochondrial transfer from MSCs and a subsequent increase in ATP production for tissue repair in alveolar epithelial cells in an in vivo mouse model of acute lung injury. These results were reproducible in other models comprising MSCs with haematopoietic stem cells^108^ or epithelial cells,^95^ with an increase in mtDNA transfer and mitochondrial content in recipient cells. Most current literature agrees that gap junctions are one of the main mechanisms for mitochondrial transfer from MSCs to the target cell,^93^ although this method of mitochondrial transfer has so far not been demonstrated in melanoma.

### Alternative mechanisms

In the three main transfer mechanisms outlined above, mitochondrial transfer is quick and unidirectional. However, Sinclair et al*.*^95^ demonstrated that, although inhibition of all these mechanisms reduced mitochondrial transfer, it did not completely prevent it, indicating the possibility that additional mechanisms exist.

Cell fusion, whereby the plasma membranes of two cells fuse and merge together whilst retaining their nuclei,^94^ is a contentious form of intercellular communication (Fig. 2d). Evidence for mitochondrial transfer via cell fusion remains scarce, as it is difficult to ascertain whether the host cell remains as a host cell after fusion.^93^ Wada et al. modelled cell fusion in vitro by developing microfluidic devices that fused paired single cells together through a microslit to form a cytoplasmic connection.^109^ They demonstrated that this cell fusion system enabled whole mitochondria to be transferred from parental osteosarcoma cells to ρ0 osteosarcoma cells and that, after transfer, the fused cells would spontaneously disconnect and recover in normal culture. Further data are required to substantiate cell fusion as a method of transfer from MSCs to cancer.

Although Guescini et al. demonstrated the transfer of mtDNA via EVs, they also showed that a significant proportion of mtDNA was free in the intercellular media.^104^ Other studies have identified the release of endogenous mtDNA, as ‘damage’-associated molecular patterns (DAMPs), into the intercellular media in response to injury and inflammation.^110^ As carcinogenesis mimics a chronic inflammatory state,^111^ it is likely that tumours secrete mtDNA into the media, identifying mtDNA secretion into the media as another potential method of mitochondrial transfer (Fig. 2e).

## Discussion

In melanomas, the TME is known to be important for conferring treatment resistance to the tumour cells. The bulk of the TME is formed by MSCs and the cells they give rise to. In contrast with melanoma cells, MSCs have stable genomes, and so offer themselves as a better potential for therapeutic targeting. OXPHOS plays a significant role in metabolic plasticity, metabolic symbiosis and the homeostasis of the high OXPHOS subset in melanoma, allowing the development of treatment resistance. MSCs ensure that melanoma cells can retain an independent OXPHOS capacity via mitochondrial trafficking to melanoma cells. Mitochondrial trafficking has been shown to be a prerequisite for continued aerobic respiration, subsequent tumour growth, metastasis and the development of chemoresistance and, consequently, inhibition of this process has been integrated into the treatment pathway for other cancers.^112,113^

In this review, we have highlighted mitochondrial trafficking as a potential target to combat the prevalent resistance to current therapies in melanoma. We also outline the need for further research into the different potential mechanisms of mitochondrial trafficking. As mentioned above, only TNT-mediated transfer has definitively been demonstrated as a means for mitochondrial transfer to melanomas; the fact that EVs and gap junctions are commonly employed for mtDNA transfer by most cancers highlights the need for further research to elucidate if these important mechanisms occur in melanoma as well, to facilitate the development of targeted therapeutics against this highly refractory disease.

## Data Availability

Not Applicable.
